# Case Report: Intraosseous arteriovenous malformation of the temporal bone presenting with drug-resistant seizures

**DOI:** 10.3389/fsurg.2025.1510821

**Published:** 2025-05-15

**Authors:** Guive Sharifi, Elham Paraandavaji, Bardia Hajikarimloo, Tohid Emami Meybodi, Jina Behjati, Yalda Nilipour, Mohammad Ali Kazemi, Esmaeil Mohammadi, Sajjad Khanbabazadeh

**Affiliations:** ^1^ Department of Neurosurgery, Loghman Hospital, Shahid Beheshti University of Medical Science, Tehran, Iran; ^2^ Skull Base Research Center, Loghman Hospital, Shahid Beheshti University of Medical Sciences, Tehran, Iran; ^3^ Neurosurgery Department, Shohada Tajrish Hospital, Shahid Beheshti University of Medical Sciences, Tehran, Iran; ^4^ Pediatric Pathology Research Center, Research Institute for Children’s Health, Shahid Beheshti University of Medical Sciences, Tehran, Iran; ^5^ Neuromuscular Research Center, Tehran University of Medical Sciences, Tehran, Iran; ^6^ Department of Radiology, Amiralam Hospital, Tehran University of Medical Sciences, Tehran, Iran; ^7^ Department of Radiology, Advanced Diagnostic and Interventional Radiology Research Center (ADIR), Medical Imaging Center, Imam Khomeini Hospital Complex, Tehran University of Medical Sciences, Tehran, Iran; ^8^ Department of Neurosurgery, Tehran University of Medical Sciences, Tehran, Iran; ^9^ Division of Vascular and Endovascular Neurosurgery, Firoozgar Hospital, Faculty of Medicine, Iran University of Medical Sciences, Tehran, Iran

**Keywords:** arteriovenous malformation (AVM), case report, intraosseous, seizures, surgery, temporal bone arteriovenous malformation (AVM), temporal bone

## Abstract

Intraosseous arteriovenous malformations (AVMs) are rare conditions characterized by the development of AVMs within bones, leading to a variety of symptoms. Temporal bone AVMs are exceedingly uncommon, as evidenced by the small number of reported instances in the medical literature. A 19-year-old male patient presented with persistent seizures that were unresponsive to pharmacological treatments administered over three years. Imaging revealed a non-enhancing lesion measuring 31 × 22 mm in the right temporal lobe, originating from the middle fossa and the right petrous apex. The patient underwent successful surgical resection of the lesion, with no intraoperative complications or postoperative neurological deficits. Diagnosis of AVM was made after intraoperative pathological investigation. Follow-up examinations showed a complete resolution of the patient's seizures. The management of these lesions is challenging and it requires personalized approaches, which depend on clinical presentation, lesion location, progression, and the patient's overall condition.

## Introduction

Arteriovenous malformations (AVMs) are rare vascular lesions that can develop anywhere in the body, though they predominantly occur in the head and neck regions (1, 2). These benign lesions result from abnormal connections between arteries and veins and generally present as masses, similar to tumors (1, 2). Without the buffering effect of capillaries, these lesions tend to enlarge over time and due to blood flow deprivation to surrounding tissues, lead to ischemic changes (2). This network of vessels, known as a nidus, is extremely fragile and prone to bleeding, stemming from the direct, high-pressure connections between arteries and low-pressure veins (2)

Intraosseous AVMs, which develop within bones, are exceedingly rare and present with a diverse range of clinical symptoms (2). These AVMs are most frequently found in the craniofacial area, particularly the mandible (3), and have also been reported in other bones, including the spine, tibia, femur, and humerus (4). Typically, these cases manifest as excessive, recurrent hemorrhage following events such as dental eruptions or surgical extractions (3).

Reports of intraosseous AVMs in the temporal bone are particularly scarce (3, 5, 6), a region where the complex anatomy—characterized by intricate bony structures and narrow canals—poses significant challenges for diagnosis and management (5). The purpose of this work is to present the first documented case of a temporal bone intraosseous arteriovenous malformation (AVM) associated with drug-resistant seizures in a 19-year-old male. We describe the clinical presentation, diagnostic challenges, and successful surgical management of this rare condition, highlighting the complexity of dealing with AVMs in the intricate anatomy of the temporal bone.

## Illustrative case

A 19-year-old male presented with a 3-year history of treatment-resistant epilepsy. His seizures were characterized by visual auras and surreal imagery that were triggered by auditory stimuli. Despite these manifestations, he remained fully conscious and aware of his surroundings throughout the episodes. The patient also reported associated symptoms, including persistent headaches, nausea, and episodic gastrointestinal distress.

The unpredictable nature of his seizures initially led the patient to suspect memory issues. However, his symptoms worsened over the 3 months prior to surgery, marked by shortness of breath, bruised lips, and facial flushes.

Following consultations with both a psychiatrist and a neurologist, he was diagnosed with a seizure disorder. Treatment with carbamazepine was initiated, which only partially alleviated his symptoms. Given the resistance to pharmacotherapy, he underwent further diagnostic evaluation.

**Figure 1:**
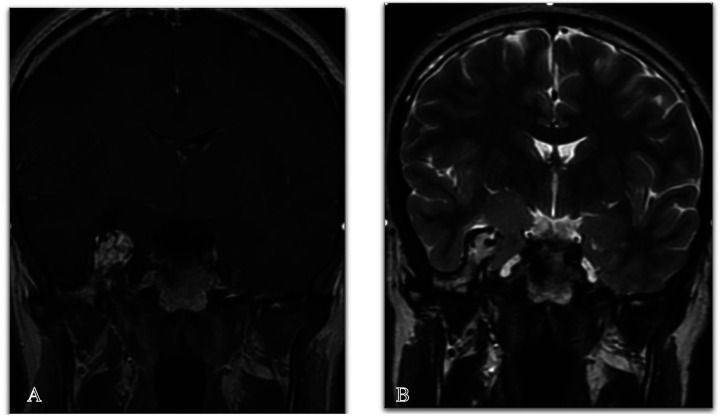
Coronal MRI images, post-contrast, show T1 sequence **(A)** and T2 sequence **(B)**, revealing a large lesion in the right sphenoid bone with intracranial epidural protrusion and an internal flow void, indicative of an AVM.

On physical examination, he showed no neurological abnormalities. T1-weighted magnetic resonance imaging (MRI) revealed a non-enhancing lesion measuring 31 × 22 mm located in the right temporal lobe, extending from the middle fossa to the right petrous apex (Figure 1). T2-weighted MRI also showed a large lesion in the same site with intracranial epidural protrusion and internal flow void. At this stage no definite diagnosis was made and vascular lesions were not suspected. For this, angiographic investigations were not performed.

**Figure 2:**
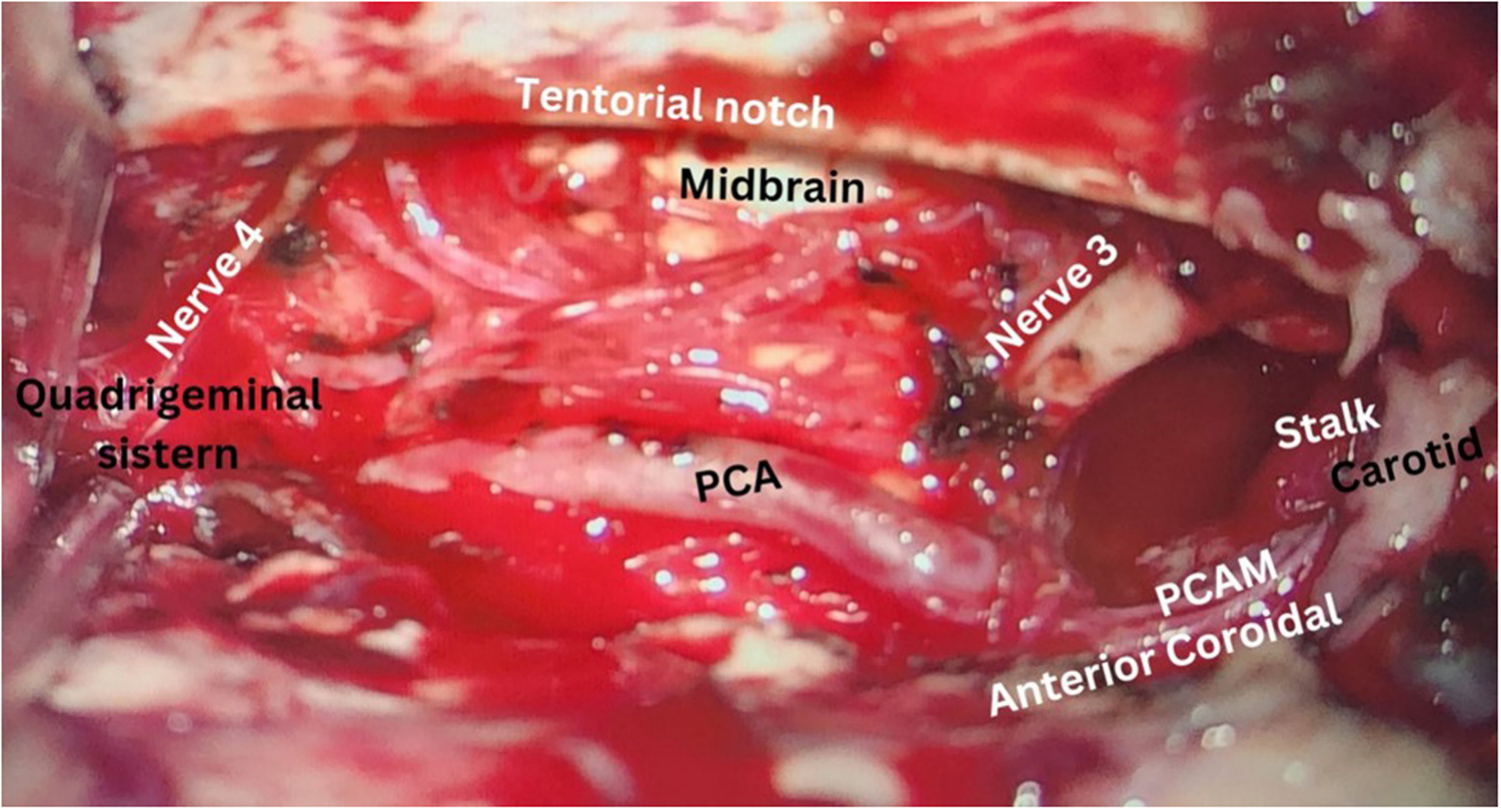
Post-resection view showing gross-total resection of the intraosseous AVM.

**Figure 3:**
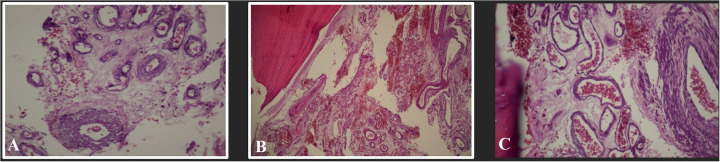
**(A–C)** Histopathological microscopy (hematoxylin & eosin staining) of the lesion showing proliferative epithelial cells, fibrous tissue, with vascular structures and endothelial cells interspersed among the bone trabeculae indicative of an intraosseous AVM.

**Figure 4:**
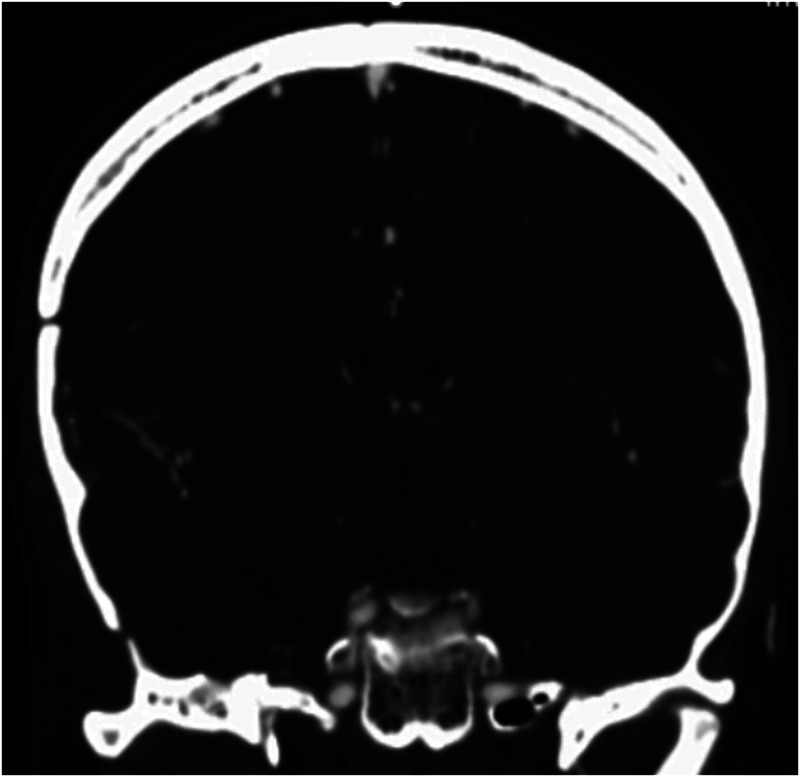
Shows a postoperative coronal brain CT angiography of the patient. A right-sided temporal craniotomy is visible. There is a defect and mild irregularity in the superior cortex of the sphenoid bone resulting from surgical resection of the AVM. The tumor and its feeders are completely resected.

To alleviate the refractory seizure, the decision was made to proceed with surgery without a certain diagnosis. The patient underwent surgery three and a half months after his seizures became resistant to treatment. Gross investigation of the mass was a vascular lesion in the temporal bone and arteriovenous malformation was suspected (Figure 2). Intraoperative sampling of the lesion was done and sent for histopathological examination which showed features indicative of an intraosseous arteriovenous malformation (i.e., proliferative epithelial cells, fibrous tissue, with vascular structures and endothelial cells interspersed among the remaining bone trabeculae) (Figure 3). Postoperative computed tomography angiography (CTA) showed gross total resection of the tumor and its feeders (Figure 4).

The patient experienced an uneventful recovery. Following surgery, his seizures were completely controlled, significantly enhancing his overall quality of life. In follow-ups he was seizure-free and had no neurological deficits.

## Discussion

We reported a case of an intraosseous arteriovenous malformation of the temporal bone presenting with drug-resistant seizures. Arteriovenous malformations (AVMs) are dynamic vascular anomalies that usually develop before adulthood, most commonly during adolescence (4). Intraosseous AVMs, a rare clinical entity, most frequently affect craniofacial bony structures (3). Following craniofacial involvement, reports have documented these lesions in the humerus, tibia, femur, and spine (4). They usually manifest in adults, with the highest occurrence in individuals in their fifties (7). In one-third of cases, bony alterations such as distortion, destruction, hypertrophy, hypoplasia, sclerosis, and osteopenia are observed (5). Intraosseous AVMs, noted for their high-flow shunting, tend to progress aggressively and are more likely to result in severe complications compared to other congenital vascular malformations (8) Studies indicate a greater risk for both spontaneous and post-surgical bleeding in these cases (2, 4). The etiology of intraosseous AVMs remains unclear, but disturbances in embryological differentiation, possibly linked to genetic factors, have been proposed as likely mechanisms (2).

Intraosseous AVMs exhibit a broad range of clinical manifestations that vary according to the lesion's location (2). Only a few cases of temporal bone intraosseous AVMs have been described in the literature (3, 5, 6). In this case report, we discuss a 19-year-old male patient who presented with persistent seizures despite three years of pharmacological treatment. Gokce et al. reported a case of Intraosseous AVM developed in the petrous portion of the temporal bone that manifested by intracranial hemorrhage (5). Nagy et al. reported a case of vascular malformation in the right temporal bone and greater sphenoid wing that manifested with the right-hand tremor (6). Seizure is the second most common clinical manifestation in the setting of AVMs and occurs in 20%–45% of individuals (9). The seizure mechanism in AVM is multifactorial, including directly due to hemorrhage and hemosiderosis or secondary to vascular steal, peri-nidal edema, nidus size and location (9). To our knowledge, this is the first reported case of an intraosseous AVM in the right temporal bone presenting with drug-resistant seizures accompanied by visual aura.

Due to the radiological similarities between AVMs and other lesions, radiological evaluations alone cannot confirm an AVM diagnosis (7, 8). Intraosseous AVMs typically appear on plain radiographs as multiple smooth osteolytic or lucent serpiginous lesions affecting the cortex and medulla (8). Computed tomography (CT) scans provide detailed views of soft tissue extension, the feeding artery, shunting points, the draining vein, and bone involvement (8). Common CT findings for intraosseous AVMs include multiple small osteolytic lesions or a single large cavitary lesion in the medulla, which may or may not involve cortical destruction (8). CT is more effective than MRI in detailing cortical changes (8). MRI is essential for outlining the spatial relationship between AVMs and surrounding structures (8). In most MRI sequences, intraosseous AVMs show signal voids in the cortex or medulla (8). MRI can also distinguish between high-flow and low-flow malformations based on the presence of enlarged feeding arteries and dilated draining veins (7, 8). Angiography is the preferred method for evaluating intraosseous AVMs and assists in planning treatment (8). AVMs are categorized into three types based on angiographic features: Type I (arteriovenous ﬁstula) involves one to three arteries shunting into a single vein; Type II (arteriolovenous ﬁstula) involves several arterioles shunting into a vein; Type III (arteriolovenulous ﬁstula) involves multiple arteriole-venule shunts (8, 10). All types display a nidus on imaging, representing an immature network of dysplastic vessels (7, 8). A conclusive diagnosis requires biopsy and histopathological analysis (2). The pathological features of intraosseous AVMs resemble those of other AVMs, characterized by proliferative epithelial cells and fibrous tissue commonly found around the lesion, with vascular structures and endothelial cells interspersed among the remaining bone trabeculae (2).

The treatment of intraosseous AVMs is complex and requires a team of specialists (1). Arterial embolization and surgical interventions are the main therapeutic options for these lesions (1). Regarding the complexity and location of these lesions, they are usually managed through a combination of the mentioned strategies (5). The preferred treatment for intraosseous AVMs is surgical resection, potentially combined with embolization of the primary feeding vessels (2). Limited data exist regarding the management of temporal bone intraosseous AVMs (5, 6). Gokce et al. chose embolization alone for a petrous part lesion to avoid potential hearing loss; however, the patient developed complications including facial paralysis and hoarseness (5). Risks associated with embolization can include hematoma at the puncture site, arterial dissection, migration of embolization material, stroke, and infection (5). According to the location of the lesion, we decided to perform surgical resection without the application of other therapeutic options. In our case no complications or neurological deficits were observed during the follow-up period. The patient's seizures were completely resolved following the surgery.

One limitation of the current case report is that an angiographic investigation was not conducted prior to surgery. Such imaging modalities could have aided in establishing a definitive diagnosis of vascular anomalies and lesions. However, this investigation was not performed because temporal bone arteriovenous malformations (AVMs) were not initially suspected in this patient, given their rare association with temporal lobe epilepsy. Therefore, we emphasize the importance of considering these pathologies in similar cases and recommend that appropriate imaging be employed to rule them out when indicated.

## Conclusion

Temporal intraosseous arteriovenous malformations (AVMs) represent an exceedingly rare clinical phenomenon, characterized by a diverse range of manifestations. This case report delineates the first known instance of a right temporal intraosseous AVM linked with drug-resistant seizures, which was effectively managed through surgical resection alone.

The management of temporal intraosseous AVMs poses considerable challenges due to the intricate anatomy of the temporal region. Consequently, treatment strategies must be carefully customized, taking into account the clinical manifestations, lesion location, the patient's progression, and overall health condition.

## Data Availability

The datasets presented in this article are not readily available because identifiable material of patient are used which cannot be shared. Requests to access the datasets should be directed to esmaeilmuhammadi@gmail.com.
